# Address

**Published:** 1885-09

**Authors:** Edward N. Harris

**Affiliations:** Boston.


THE
AMERICAN JOURNAL
OF
DENTAL SCIENCE

VOL. XIX. THIRD SERIES.—SEPTEMBER 1885. NO. 5.

ARTICLE I.

### ADDRESS,

(Delivered before the American Academy of Dental Science, at their 17th Annual Meeting, held in Boston, November 5, 1884.)

BY EDWARD N. HARRIS, D. D. S., OF BOSTON.

*Mr. President, and Gentlemen of the American Academy of Dental Science:*

With thankful hearts to Almighty God, the giver of all good, we meet to-day to celebrate this Seventeenth Anniversary of our Academy.

I am not insensible to the high honor that you have conferred upon me in selecting me to deliver the Annual Address before you upon this occasion, and follow in the long line of eminent practicioners who have preceded me during the years that are past. I appreciate this compliment, coming as it does from a society which bears so high and honorable a record, and in which I have felt a lively interest from its earliest organization in 1867, down to the present time; and from an association of professional gentlemen whose acquaintance and friendship I have enjoyed through many years, and for whom I entertain the highest feelings of fraternal respect.

Associations like this unite men of different opinions, and conciliate friendship among those who might otherwise have remained at a perpetual distance.

In my remarks to you to-day, I will first speak briefly of the early history and the progress of this Society, and of some of the advantages that dental societies are to the profession and to the community. I will then say something about the advancement the art and science of dentistry has made since I first started out as a dental practitioner, thirty-one years ago; and, lastly, I will call your attention to a subject which is agitating the public mind at the present time, viz.: Christian Science, or Metaphysical Healing, or what is frequently termed the Mind Cure, and which may have an important bearing upon our profession in the future.

This Anniversary measures another year in the onward march of our own developments and achievements for the profession of our choice. This day is to us a resting-place in the history of our labor and our progress. It is that future of our early hopes to which our eyes have looked, our hopes have longed, and the feeling is strong upon us now to sit down together around the old camp fire and rehearse the struggles and the triumphs of the past, and live over again the many pleasant occasions we have enjoyed, and speak of the many grand events that have taken place, and the noble work that has been accomplished during the past seventeen years in and through this American Academy of Dental Science.

It was my good fortune to be present at the opening day, October 19, 1867—a day I shall always cherish in memory—when a few of us gathered together and started this Academy into life.

This was the second dental society instituted in Boston, the first and only one preceding it was the Massachusetts Dental Society, organized in 1864, of which I was one of the original founders.

The small band of professional dentists who assembled to take into consideration the expediency of forming this Academy, met with considerable opposition, but they were men of resolute purpose, and with strong love for their profession, possessing an earnest desire for the advancement of dental science, and they felt that if such an Academy could be formed it would receive the encouragement and co-operation of a large portion of the better class of practitioners, and the best interests of the profession would be promoted, and great good would result to the public.

As we take a retrospective view of the past, and note the progress made from year to year, we shall see that the anticipations of those original members who projected this enterprise have been more than fully realized. For a while at first it was misunderstood by some, and misrepresented by others, as an exclusive and aristocratic movement; but unmindful of this, the Society moved steadily forward, doing its appointed work, and within a few years gained a firm hold on the confidence of the community, and commanded the respect of the profession throughout our entire country and in Furope.

From the catalogue issued the past year, I find that since the formation of the Academy, one hundred and twenty-four members have been admitted, including among them many dentists of eminence in America and Europe, and making a roll of membership of which this Society can feel justly proud. The names of our honored presidents and orators are familiar to you all—they need no encomium from me.

| | | |
|---|---|---|
| Elisha T. Wilson | Elected | 1867 |
| Daniel Harwood | " | 1868 |
| Joshua Tucker | " | 1873 |
| David M. Parker | " | 1875 |
| Elisha G. Tucker | " | 1877 |
| Jacob L. Williams | " | 1879 |
| Thomas H. Chandler | " | 1881 |
| George T. Moffatt | " | 1882 |

OUR ANNIVERSARY ORATORS.

| | |
|---|---|
| Elisha T. Wilson, of Boston | 1868 |
| Daniel Harwood, of Boston | 1869 |
| Joseph H. Foster, of New York | 1870 |

| | |
|---|---|
| John H. McQuillen, of Philadelphia . | 1871 |
| Philip H. Austen, of Baltimore . . | 1873 |
| W. W. Allport, of Chicago . . . | 1874 |
| Robert Arthur, of Baltimore . . . | 1876 |
| William H. Dwinelle, of New York . | 1877 |
| Charles W. Eliot, President of Harvard University . . . . . . | 1878 |
| C. A. Marvin, of Brooklyn, N. Y. . . | 1879 |
| Joshua Tucker, of Boston . . . | 1880 |
| Frederic N. Seabury, of Providence . | 1881 |
| Frank Abbott, of New York . . . | 1882 |
| Norman W. Kingsley, of New York . | 1883 |

Nearly all of the addresses delivered by these gentlemen have been published by the Academy, and copies are on file with our librarian for permanent preservation.

During these seventeen years, twenty-six of our number have been called away from earth, and have entered the land of rest and immortality. They have passed through the change which the world calls death, but which is only a transition to a higher degree of life and an ascent in the scale of existence. And it is fitting that we here pause for a moment and drop a silent tear, and speak a word of tribute and affection to the memory of those departed brothers, whose able counsel, genial companionship, and cheering words, we enjoyed at our monthly and annual meetings through many years.

Their seats are vacant here to-day—we miss them—and though their beloved faces we can no longer see, and their familiar voices we can no longer hear, yet in fond recollection we can see and hear them; and now, as this Anniversary hour comes on, we can still feel their living presence inspiring us with renewed hope and zeal in our profession, and with love for one another and charity towards all mankind. The memory of the just is sweet, and theirs will live in hallowed fragrance through generations yet to come; and the noble work they accomplished through a long series of years to elevate and adorn their profession and to advance the interests of this Academy, and the great benefit they conferred upon humanity in preventing and alleviating human suffering, will ever be acknowledged and remembered, and their names will continue to be cherished, not only by us, their surviving friends and associates, but by their grateful patients both here and in distant States and lands, and by the dental profession everywhere.

Some of them had their peculiarities, so have we all; but I will venture the assertion that no future generation of dentists will ever find within their ranks twenty-six more able and faithful laborers, or twenty-six more true and honorable gentlemen. Quite a number of them lived to a very advanced age, and they left behind them a record and an example well worthy of imitation by the young men now coming into the profession. In their declining years they had the pleasant satisfaction of seeing the science and art of dentistry and the status of the profession advancing towards the ideal that they had formed for it in the thoughts and aspirations of their earlier years.

Having briefly alluded to the past history of this Academy, and spoken of the progress that has been made and the high ground that has been reached, let us leave the scenes of recollection for the survey of the field we occupy, and the prospect that opens beyond. At our monthly and annual meetings we meet to investigate every truth upon which our art is founded, that we may aid in its advancement, and be better qualified to fulfil our duties to our patients. We meet to compare notes, to exchange ideas, to learn from each other's experience, and to refute or confirm opinions previously entertained, and thus impart to one another and to the community in which we live, useful and practical knowledge.

We meet also to renew old acquaintances, and to draw more closely those fraternal bonds which should bind us to each other and to the profession we esteem. I say to the *profession*, because being conscious of our indebtedness to it in the past, and the great progress it has made in our country, we should feel a pride in doing all we can to maintain its present prestige, and press forward to a still higher position.

One of my dear old teachers, the late Prof. P. H. Austen, in a valedictory address to the graduates of the Baltimore College of Dental Surgery, in 1853, used the following beautiful and impressive language:

"A man may redeem the follies of his youth; a science correct the mistakes of its infancy. The arts of medicine and surgery have thrown over the ignorance and misconduct of their youthful days a thick veil of noble deeds, lofty aims, profound learning, and heroic self-devotion. If to the art of dentistry, youngest in this noble band of three, that stand to minister at the altar of suffering humanity, there still clings some of the reproach of her early associations, it is not because she is more lowly born than they. The deeds of her childhood might well be the boast of riper years; they give sure promise of a maturity of which her elder sisters shall have full reason to be proud. Now, in the broad twilight of the present, to make the darkness of the past forgotten in the brightness of the future, and increase onward and upward till it shall reach the fullness of noonday splendor—this, gentlemen, is your mission."

The prophetic utterances of Dr. Austen, made thirty-two years ago in Baltimore, are being fulfilled. Since that time, dental colleges and dental schools in universities have been established in different parts of our country, and dental societies have been instituted in every State of our Union, and also in Europe—and all these in the aggregate have done a mighty work in the education and elevation of the profession. The standard of general dental practice has improved at least fifty per cent. since the organization of dental societies.

Associated effort for the accomplishment of any purpose is one of the greatest promoters of progress.

Dr. C. E. Francis, of New York, at one of our anniversary meetings some years ago, very truly said, " That dental societies are the very backbone of the profession. It is at such gatherings that the members bring their best thoughts, and keep up the spirit that is inculcated at the colleges," The first dental society in America, and I may say in the world, was organized in New York City in 1840, as a national association, under the name of the American Society of Dental Surgeons. I am happy to be able to say that quite a number of the members of that old distinguished pioneer society became members of this Academy—a few of them still survive—one of these seniors we rejoice to see present here to-day—a gentleman full of years and of honors, and one who took an active part in the formation of this Academy, and who has ever since favored us with his presence and words of good-cheer and encourogement at all of our annual meetings, and at most of our monthly meetings. I refer to one whom you all delight to honor, Dr. E. G. Tucker, of this city.

When I began the study of dentistry thirty-three years ago, there was not a dental society in the New England States, and but six in the whole United States. There were but two dental colleges actually in operation. Two others procured charters during that year. There are now twenty colleges and more than one hundred State and local societies and two national associations in our country alone. besides several valuable dental journals, issued monthly, and many standard text-books and scientific works upon the different branches of our art, published by able authors. Showing what remarkable progress has been made within the compass of half an ordinary life-time.

To New Hampshire belongs the honor of starting the first society of dentists in New England, which was organized at Concord in 1853, under the name of the New Hampshire Dental Society. In the fall of 1854 the Vermont Society of Dental Surgeons was organized at Montpelier. Ten years later, Boston awoke out of its long sleep, and started the Massachusetts Dental Society, in 1864, and elected for its first president an old and eminent practitioner of this city, the late Dr. N. C. Keep, who continued his interest in that society up to the time of his decease. The Massachusetts Dental Society has done a good work for the profession and the community. It has passed through some vicissitudes, but it still lives.

During the past ten or twelve years two or three other societies have been formed, so that Boston is now well supplied. It seems to me that instead of dividing the forces so much, the wisest course for one of the younger societies to pursue would be to unite with the American Academy of Dental Science by a transfer of membership, and consolidate their forces and interests with ours. By adopting this plan, all would be mutually benefited; new life and new strength would be infused into this Society, and it will move onward, strong and progressive, towards a brilliant future.

As the reputation of a person is determined by his acts, so the reputation of a profession or a soeiety is determined or established by its acts, and by the abilities and activities of its members. There is a great deal of ability here, among our members, and all we need is a little more earnestness and activity in the work of the society. It has been said that a man or a woman can become lazy mentally as well as physically. We should each do something to leave its imprint, either by excelling in what has baen done, or by taking some advanced step. Devotion to our profession is the secret of success. Nothing great was ever achieved without enthusiasm. We should cultivate self-reliance, and have a strong and high appreciation of faith as to our own God-given powers, which will enable us to work as others work, learn as others learn, and succeed as the best. The poet spoke most truly when he said:

> "Our doubts are traitors,
> And make us lose the good we oft might win,
> By fearing to attempt."

Our meetings afford an excellent opportunity for active work, and every member should regard it as a duty to present his views upon the various subjects that may come up for discussion. Differences of opinion may exist, but their free expression, coupled with a kindness of consideration for those who differ, will advance instead of retarding the interests of science. All the professions and many of the principal occupations of the present day, have their associations for mutual improvement and benefit, and it well becomes our specialty to be not less active. And let us bear in mind that the members of the dental societies, acting in co-operation with the faculties of our dental colleges, are together, the educators of the future profession.

In the spirit of inquiry and investigation which prevails, it is gratifying to observe that enthusiasm is not permitted to run away with judgment in any particular branch, so that the members of these societies are for the most part adopting a more conservative mode of practice than that which prevailed fourteen or fifteen years ago, when hard gold and mallet filling were talked up so loudly as the *only* practical methods. We thoroughly test the alleged modern improvements, many of which are truly valuable, and many are worthless, and we adopt only those that *prove* to be useful and practical, and save time and labor, while the rest we discard for a return to some of the methods the old fathers taught us which proved so eminently satisfactory during their long and successful experience in preserving the natural teeth.

Dental societies now exist in every State of our Union, and several have been organized in Europe.

"Thus the dental graduates of the colleges and the universities are getting into one vast associated fraternity, and establishing an efficient professional organization, with its policy adjusted in every direction to the cultivation of the science, and to the regulation of the conduct of the profession; thus forming a standard professional sentiment, and through its agency, raising public opinion into a supporting conformity."

As one of the senior members it may not be assuming too much for me to suggest that the Academy be not too exclusive in the admission of new members. Our membership has increased very slowly during the past few years. This can be attributed mainly to a greal lack of effort on the part of our members, myself included, to interest others and induce them to join the Academy. It is well to be cautious and choice in our associations with other practitioners, but not fastidious. During this coming year, let each one of us make a stronger effort to bring in some new members, and especially from among those who have recently graduated and are yet young in experience and practice, for to the young men we must look for the future progress and devolopment of our profession, and the continuation and success of this Academy. In this way we shall render our meetings more interesting, and enlarge and improve our opportunities for doing good unto others.

There have been from time to time during past years; some practitioners, not many, who seem to chafe under the custom which prevails of being termed dentists, and a few have even advocated the dropping of the word *dentist* from our nomenclature, and substituting some other name. Various names have been suggested to take its place. In the earlier years the phrases surgeon dentist, surgical and mechanical dentist, were sometimes used—in later years operative and mechanical dentist, and the words dentician, dentificier, dentologist, oral surgeon, oralist and orist, have been suggested and by some used. Now the question is which one shall the profession adopt, and have registered in the new dictionary of the future.

As the word dentist came so honestly from the Latin word dens—a tooth, and is so universally used at the present day, it seems to me that the only course to pursue is to continue the term *dentist*, and let the profession through its proper representatives, communicate with lexicographers, and prevail upon them to give in their future editions of dictionaries a more extended and comprehensive *definition* of the word than they have in the past.

The definition given in Harris's *Dictionary of Dental Science* is good, viz.: "One who devotes himself to the study and treatment of diseases of the teeth and their connections, and which at the same time embraces the prosthesis, or replacement of the loss of these organs, with artificial substitutes." In speaking upon this topic, Dr. Austen said, "We do not say surgical aurist or ocular surgeon, for the aurist and the oculist treat the medical as well as surgical diseases of their respective organs; therefore to him who must bring medical, surgical, and mechanical skill into such constant and harmonious exercise as our profession, our specialty, requires, that term is most appropriate which will at once express all its duties. This you will find in the comprehensive name Dentist. I would then advocate the discarding of all phrases of partial significance, and let this be the name which it shall be our delight to honor. Let us put away from us any insinuation of inferiority which would be implied by prefixing to it or substituting for it any other term, and acknowledge the right of none to its adoption, who are not qualified in all that it comprehends."

I appreciate the high motives of those gentlemen who advocate the terms oral surgeon and oral science in the place of dentist and dental science. They do so because so many of the followers of our art have degraded the profession, and brought a reproach upon the name of dentist by their ignorance and malpractice, which has caused many men of science and education in other professions to place a low estimate upon dental practitioners generally—but the community is becoming more enlightened, and is beginning to discern between the educated and the uneducated dentist, and to discriminate between the true and false, the scientific and the pretender.

If we drop out the term dentist, then we shall be obliged to drop all words of a kindred nature which have the same derivation, viz: dentistry, dental surgery, dental medicine, dentition, dentation, denticulate, dentiform, dentoid, denture, and some others, necessitating a complete change in our dental vocabulary. I trust those gentlemen who proposed this change, and who continue to advocate it, will yet be convinced of its utter impracticability. Like the dental profession, the ranks of the medical profession are infested with charlatans, but we never hear of their advocating the discontinuance of the term physician because of its free use by the class mentioned. Let us then retain our title of dentists, and each one strive to elevate it, and we need not fear that the name will degrade us. Our profession is rapidly redeeming the past, and proving her right to a high seat among the sciences, which she claims. Men whose talent and education would adorn any calling, she now numbers by hundreds, and I might say thousands.

A few words in regard to maintianing the dental laboratory. There are some, not many, who are inclined to exclude from the list of essential duties such as are in any wise mechanical. They seem to view the laboratory work, the making of artificial teeth and plates, as too mechanical, and therefore somewhat degrading, now if the exercise of mechanical skill is degrading, then our whole art must be so, for nearly all of our operations depend for success upon this same species of skill. Is it a higher order of work to extract a tooth than to replace one? I admit that handling gold-foil and saving the natural teeth may be an operation of more importance to our patients in the aggregate than making and fitting artificial teeth, which restore to the denuded arch beauty and usefulness—but are they not both honorable operations, often requiring the highest skill and ingenuity? Newton made the telescope, Fahrenheit the thermometer, Angelo the statue, Raphæl the landscape. "When the brush of the painter or the chisel of the sculptor shall be thought to tarnish the genius that hides them; then, but only then, may we look down on the tools wherewith we must work out the high and useful purposes of our art." Because the laboratory work has not of late years kept pace with the improvements in operative dentistry, but has been degraded down by poor work and cheap prices, and is to a great extent in the hands of the unscrupulous and unskillful, we *should not forsake* this department of our art, but take a stronger hold of it, rescue it, and raise it up again to the respectable place it once occupied.

Let us then maintain our laboratories as indispensable adjuncts to our operating rooms, and teach our students the use of tools as well as instruments, and how to use the dentists' lathe as well as the dental engine, how to restore the human face divine as well as how to preserve the natural teeth. Let the office and laboratory go on kindly together, with no idea of a separation, or a divorce, as some have advocated.

Times will not permit, neither would it be advisable in an address upon an occasion like this, to enter into any special discussion of the effects of food and drink upon the teeth; but I would like to call your attention to an able paper upon "Man and his Teeth," written by Dr. E. W. Foster, of this Academy, and published in the *Dental Cosmos*, Volume 18, wherein he speaks of the influence the water we drink has upon the teeth. And I would here say that my experience and observation during my long practice as a dentist, enables me to fully coincide with the views of Dr. Foster on this subject. It is necessary that the whole paper should be read in order to get at an intelligent understanding, or a fair estimate of the points he has there so well presented and proved. I can give here only a short extract from the paper. He says:

"Water has an almost inestimable influence on the teeth as well as upon the rest of the body. In fact, we have data to prove that children having plenty of water called 'hard water'—*that* water holding in solution a greater proportion of nutritive salts than the so-called 'soft water,' will have good teeth almost invariably; while our modern systems of water-works of lake and river water evaporated in reservoirs, and holding much organic matter in solution, and also the system which prevails to a large and increasing extent in many of our interior towns, of building tanks or cisterns in cellars to catch rain-water for drinking purposes, and in each case being 'soft water,' will have, as in the nature of the article supplied, sooner or later, a pernicious influence on the teeth of those compelled to use it for food and drink.

"The Old Oaken Bucket is not alone a sentimental myth, but a practical reality. And the old well-sweep of our boyhood, or the clear, hard water spring from the hill-side that gave its priceless supply to the bony and dental systems of its partakers; that water so healthful and relishing, that has inspired so much genuine poetry and reflection, and that has such life-giving power and so beautifully reminds us of that 'well of water which springeth up into everlasting life,' is personally and intimately practical to us all.

"In a word, I would say 'hard water' for culinary and drinking purposes, and 'soft water' for washing and other uses. Water is Nature's universal food. It forms a large bulk of all we eat and drink, and is a large part of all we are ourselves. Here we see the most fluid and health-giving of all soft foods, furnished by nature herself; and I will show you the best teeth where the water for family use has been best for sustaining adult organization, and the growing wants of the young. And this water comes from old, deep, and mossy wells—wells holding in their clear cold depths the solution of certain metallic riches of the earth, riches that man must have, and that nature here so kindly and sagaciously provides. Or, again, the springs by the wayside, into whose tiny basin a liquor fit for immortals is distilled from rock mountain sides, and nature says to man: 'Drink, O mortal traveler; then pursue thy way with strong limb and purpose, cast a smile upwards to thy ancient mother, and let the light of thy face pale before the iridescent glow of thy fair and beautiful teeth.' In early childhood, milk, another fluid food of the most vital importance, rears and develops the teeth. Air, light, and happy thoughts, and rays of hope and laughter, and good fellowship, are also foods for the teeth."

And now lastly, but not least, I desire, gentlemen, to call your attention to a subject in which I have of late taken a deep interest. I refer to the new dispensation in the healing art, or I might say the ancient practice revived, of Metaphysical Healing or Christian Science; a subject that is destined as it shall become known and understood to be of great benefit to the race, and one that is to be of mighty importance to our profession in the future, in allaying the *fears* and *pains* of our patients while undergoing operations in dentistry, and in preventing any unfavorable after effects, and also in preparing them for the operation by removing or lessening the *dread* which most persons feel when contemplating a visit to their dentist. The understanding of this science will also enable us as dentists to operate with greater ease and less personal fatigue, which is an important consideration to us in the laborious and wearisome vocation of the dental practicioner, often so exhausting to the nervous system. You are doubtless aware that this subject is attracting much attention in this community, and in other portions of our country at the present time, and also in England, where it is causing a very spirited and even acrimonious discussion. It is a noteworthy fact that two societies have been recently organized to investigate, in general terms, the influence of Mind in Nature—one called the English Society of Psychical Research, and the other the American Society of Psychical Research. These societies embrace within their membership many eminent names in scientific and philosophical circles in Great Britain and the United States. In the *Dental Cosmos* I observed a notice of a work recently published, entitled *Illustrations of the Influence of the Mind upon the Body in Health and Disease, Designed to Elucidate the Action of the Imaginatian*, by Daniel Hack Tuke, M. D., L. L. D., Fellow of the Royal College of Physicians, London. In this volume, containing four hundred and eighty-two pages, the author, taking as his text the saying of John Hunter, "There is not a natural action in the body whether involuntary or voluntary, that may not be influenced by the peculiar state of the mind at the time," has formulated the generally accepted facts of physiology and psychology as they bear on the question of the influence of the mind upon the body. He has collected into one volume, from all sources at his command, authenticated facts illustrative of this influence, and supplemented them by instances which have come under his own observation. This is considered a superb work, and should be in the library of every dental practitioner.

In a recent discourse upon the Mind Cure, delivered by the Rev. Dr. C. A. Bartol, which has since been published in pamphlet form, he said: "In using the terms *Metaphysical* or *Christian Science*, the new practice disowns aught magical or lawless in its belief or procedure, appeals to common sense and common experience to attest its claims, and plants itself upon the base the Bible builds on, fact and principle in human nature; not despising but confirming God's recorded or unwritten revelation, coming like Christ, not to destroy but to fulfil." What a beautiful tribute to this science from one of Boston's oldest and most learned divines. He further said: "The attenuation of medicine which has worked so well may end in its annihilation," and he "greets the new departure which lays the stress on mind," and adds, "let us not with cast-iron prejudice reject whatever agrees not with our prepossessions."

During the past three years I have devoted considerable time to the investigation of this science. And it may be truly designated a *science*, for it is founded on a principle that can be demonstrated and proven. The power of mind over the body is as yet but little known and understood. Dr. Arthur T. Buswell, Christian Scientist, of Boston, in a recent communication to the press upon Metaphysical Healing said; "Probably no subject of reform has received so much able thought in both the old and new world, as the relation of science to religion; but hitherto the sanative and reformatory qualities of Divine Truth and Love have been practically excluded as intelligent remedial agents, by the doctors of both mind and body."

There has been much written upon the Science of Mind or Soul, and the Divine Law of Cure, and on Mental Healing, Faith Cure, Mind Cure, and Mental Medicine, and some of them are works of excellent merit; but from quite an extended examination and research and inquiry, I find that the science and laws of purely mental healing and their method of application through spiritual power alone, were discovered and brought out to this age by a Woman—a lady of rare intelligence and refinement, and of high Christian character, Mrs. Mary Baker G. Eddy, now President and leading Professor of the Massachusetts Metaphysical College, 571 Columbus Avenue, in this city. This institution was regularly chartered by the Legislature of Massachusetts in 1881, under Governor Long's administration, and as set forth in the annual announcement: "Male and female students are here taught metaphysics on a purely practical basis, to unfold the resources of unfathomed mind, to impart a thorough understanding of mental science, to restore and preserve health, and to elevate man physically, morally, and spiritually, and thus restore to the race hope, health, and the lost science of Divine or Christian Healing."

This is the first legally founded and the only thoroughly metaphysical college in the world. It has a powerful ally in the Christian Scientists' Association, a large and influential organization, whose membership is made up of graduates of the College, actively engaged in healing; an earnest band pledged and working together in a common cause of humanity and love. This Association is the only body of organized metaphysicians in the world.

The following account of the circumstances through which Mrs. Eddy discovered this principle of healing and the metaphysical science that governed it, is taken from the *Christian Science Journal*, a monthly magazine published by the Association, and the narrative is replete with interest:

"Some eighteen years ago this woman, who was a practising homeopathic physician, had her attention called to the great influence which the minds of her patients exercised over their bodily condition. She was a thoughtful woman with an independent and original mind, which could not be limited to the conclusions of other minds. Following this line of investigation thus indicated, experimenting with doses of pure water, when a favorite remedy was expected by the patient, and gaining exactly the desired result; in other cases bringing her mind to bear on the disease, and restoring the patient to health, unaided by drugs or the imagination, she gradually became convinced that the power of mind over matter was an almost undiscovered and a wholly incalculable force. Later, she received injuries by an accident which her attending physician and surgeon pronounced fatal, and said she could not survive over three days. Her limbs were paralyzed, and her suffering great. The third day was the Sabbath; her clergyman visited her before services, prayed with her, and said farewell. She asked him to call after meeting. He replied by asking her if she knew the fatal nature of her injury, and that she was sinking, and might not survive through the day. She replied that she knew it all, but had such faith in God she thought he would raise her up. After he left, she requested to be left alone; the room was full of people, but they all passed out. She then gave her mind intently to the New Testament account of Jesus' healing the withered hand on the Sabbath day. As she read, suddenly a great change came over her; her cold, immovable limbs became warm and full of life, the internal agony ceased, her strength came instantaneously, and she arose from her bed and stood upon her feet, a well woman. The clergyman called after services, and she met him at the door, and that day prepared the evening meal for the family. Both her clergyman and physician were astounded at her recovery. There are persons living who can attest to the above facts.

"She says; 'For three years after this, I sought day and night for the solution of the problem, How was I cured? I searched the Scriptutes, reading nothing else, not even a newspaper—kept aloof from society, and devoted all my time and energies to discovering a rule for that demonstration. I knew its principle was God, and thought it was done according to primitive Christian Healing by a certain action of mind on the body, through a holy, uplifting faith; but I wanted to find the science that governed it, and by the help of God and no human aid I did find it.'"

This she claims to have discovered and to have demonstrated by the healing of hundreds of people, many of whom have been pronounced incurable by the best physicians.

Mrs. Eddy has labored with tongue and pen to found this system, and for the last sixteen years has taught this theory to others in so far as their minds were capable of receiving. She has printed and published two volumes, entitled *Science and Health* with a *Key to the Scriptures*—in which the principle underlying this science is explained. This is the most remarkable book on Health and Mental Healing that has ever been placed before the public. It is having a great sale, and has already reached the twelfth edition. The author claims that the methods of healing which she has introduced to this age, are those of Christ and His Apostles. Jesus commanded his disciples to "Go into all the world and preach the gospel and heal the sick." This divine command is just as binding upon Christ's ministers and disciples of to-day as it was in the days of the Apostles. He also told them: "He that believeth on me, the works that I do shall he do also; and greater works than these shall he do, because I go unto my Father."

"And these *signs* shall follow them that believe; in my name shall they cast out devils; they shall speak with new tongues;

"They shall take up serpents; and if they drink any deadly thing it shall not hurt them; they shall lay hands on the sick, and they shall recover."

Mrs. Eddy says the principle of Mental Healing is Divine and Eternal; but the application of it to heal the sick has been lost sight of, and required to be again spiritually discerned and its science discovered, that men might retain it through the understanding. She begins her book on Science and Health as follows: "At the Oxford University, England, a prize of one hundred pounds has been offered for the best essay on Natural Science that refutes the tendency to attribute physical effects to physical causes rather than a final spiritual cause.

"A demand for metaphysics expresses the wants of the race. It is the one question to be considered, for it relates more intimately than all others to the progress of mankind. The age seems ready to verge upon this subject, to think briefly on the supremacy of spirit, and to touch the hem of its garment and be made whole. The utter control of mind over body is no longer a question with us; we have gained its proof by demonstration, and have reduced our discoveries to a system, stated the principle upon which it is based, and the rules for applying metaphysics to the treatment of disease.

"After a careful examination of the discovery in metaphysics that mind governs the body not in part but wholly, we submitted our metaphysical system of treating disease to the broadest practical proof. Our theory has gradually gained ground, and established its own proof whenever it has been employed honestly and under circumstances that permitted its demonstration, as the most effectual curative agent in medical practice.

"As time is working wonders in the world we call material, the swift pinions of thought are soaring to the realm of the real, the first cause of all things. A material basis whence to deduce all that is deemed rational is yielding slowly to a metaphysical basis of reasoning, changing from matter to mind to discover cause and explain effect. The honored materialistic philosophers, Professors Tyndall, Huxley, Agassiz, and others, appear to challenge to final combat physics and metaphysics; and at this Utopian period, like the shepherd-boy with his sling, woman goes to battle with Goliath."

The power of mortal mind over its own body is great, its action to destroy the body, reversed, will restore health. John Hunter, the great surgeon and anatomist, said: "As the state of the mind is capable of producing a disease, another state of it may effect a cure."

A. Bronson Alcott, after perusing the books, *Science and Health*, wrote the author as follows: "The profound truths which you announce, sustained by facts of the immortal life, give to your work the seal of inspiration—reaffirm in modern phrase the Christian revelations. In times like these, so sunk in sensualism, I hail with joy your voice, speaking an assured word for God and immortality, and my joy is heightened that these words are of woman's divinings."

Many persons, and among them several of my acquaintance, have recovered their health from reading these books. All science must be thoroughly taught to be understood, and understood to be demonstrated—so you may not at first accept all the statements and conclusions found in these volumes, and you cannot fully understand them without further explanations and personal instructions from the author, who is eminently qualified as a teacher, and possesses a remarkable faculty of imparting her knowledge to her students. I believe she has been selected for this high mission, as the one from among all the millions, because of her peculiar fitness for this grand work of leading the way, and introducing this great subject to this age and to the world. Hundreds of hopeless invalids are constantly being restored to health by her methods, and many dissipated, degraded men and women are being brought to lives of sobriety and virtue. For this science not only heals the sick but reforms the sinner at the same time; so we can but give praise and God-speed to this noble work which as it shall become known and understood must prove of untold benefit to this and future generations.

Since the opening of the college mentioned, and during the previous years that Mrs. Eddy has been engaged in teaching this science, nearly one thousand students have availed themselves of her system of instruction. Some of them have studied merely for the benefit of their own health and that of their families, but most of them are now engaged in practising this new art of healing as a profession, in this and in other portions of our country. They practice under the title of C. S., Christian Scientist, and they employ neither drugs, magnetism, manipulation, nor will-power, and are strongly opposed to the notions of modern Spiritualists, and are unbelievers in clairvoyance. Their fundamental ideas are that all substance is spirit, and Spirit is God, that man is God's idea, and hence he is immortal—that good is eternal, evil an unreality, and the punishment for sin is limited and remedial. Spirit is God, and God created man in his own image and likeness; hence, man is spiritual and not material. God did not create sin, sickness or death. Whatever is of God's creation is good—God is Life, Truth, Love and Harmony. The *unreality* of evil, whether sin, sickness, sorrow, or death, is one of their strong points in healing the sick. They take the opposite thought to what their patients believe through their personal senses. They treat error with Truth, discord with Harmony. Good is the opposite to evil—Good is the real, evil the unreal. Health is the opposite to sickness and opposite to death, Harmony is the opposite to discord—Sorrow is not the master of joy—Joy is the real, and is the stronger power, and must overcome sorrow. Fear is not the master of Courage—Courage is the stronger power and must conquer fear. It is the *fear* of sickness or disease, either in the conscious or unconscious thought, that brings it out through the mortal mind upon the body.

Page(s) missing

